# INVOLVEMENT OF CATECHOLAMINES IN THE MYOCARDIUM OF RATS SUBMITTED TO EXPERIMENTAL MODEL OF PORTAL HYPERTENSION

**DOI:** 10.1590/0102-672020180001e1383

**Published:** 2018-08-16

**Authors:** Antonella VINHOLI, Marília Da Cruz FAGUNDES, Danieli Cristina PIGOZZO, Fernando Bermudez KUBRUSLY, Luiz Fernando KUBRUSLY, Camila Aparecida Moraes MARQUES

**Affiliations:** 1 Faculdade Evangélica do Paraná and Institute of Medical Research/Post-Graduation in Principles of Surgery;; 2 Department of Physiology, Universidade Federal do Paraná;; 3 Denton Cooley Institute;; 4 Health Sciences Sector, Universidade Federal do Paraná, Curitiba, PR, Brazil

**Keywords:** Portal Hypertension, Portal vein, Ligation, Tyrosine 3-monooxygenase, Sympathetic nervous system, Hipertensão portal, Veia porta, Ligadura, Tirosina 3-mono-oxigenase, Sistema nervoso simpático

## Abstract

**Background:**

The role of autonomic nervous system in the development and maintenance of portal hypertension is not fully elucidated. It is known that the gene expression of norepinephrine in the superior mesenteric artery varies with time, and it may contribute for splanchnic vasodilation and its consequent hemodynamic repercussions. It is still not known exactly how the adrenergic expression behaves at the heart level in the initial stages of this process.

**Aim:**

To evaluate the immunohistochemical expression of the enzyme tyrosine hydroxylase (tyrosine 3-monooxygenase), involved in the synthesis of norepinephrine, in the myocardium of rats submitted to partial ligation of the portal vein.

**Methods:**

Twenty-four *Wistar* rats were divided into two groups: Sham Operated and Portal Hypertension. The partial ligation was performed in the Portal Hypertension group, and after 1/6/24 h and 3/5/14 days the animals were euthanized. Immunohistochemical analysis was performed to quantify the expression of the stained enzyme using the ImageJ program.

**Results:**

The Portal Hypertension group expressed percentages between 4.6-6% of the marked area, while the Sham Operated group varied between 4-5%. Although there was no statistical significance, the percentage stained in the Portal Hypertension group followed an increasing pattern in the first 6 h and a decreasing pattern after 24 h, which was not observed in the Sham Operated group.

**Conclusion:**

The expression of noradrenaline in rat myocardium during the first two weeks after partial ligation of the portal vein, with tyrosine hydroxylase as marker, did not show differences between groups over time.

## RESUMO

**Racional:**

O papel do sistema nervoso autônomo na hipertensão portal não está completamente elucidado. Sabe-se que, nessa condição, a expressão gênica da norepinefrina na artéria mesentérica superior modifica-se com o tempo, podendo ser importante contribuinte para a vasodilatação esplâncnica e suas repercussões hemodinâmicas. Apesar dos estudos sobre as repercussões cardiovasculares na hipertensão portal, ainda não se sabe como a expressão adrenérgica se comporta a nível cardíaco nas etapas iniciais desse processo.

**Objetivo:**

Avaliar a expressão imunoistoquímica da enzima tirosina hidroxilase (tirosina 3-mono-oxigenase), relacionada à síntese da norepinefrina, no miocárdio de ratos submetidos à ligadura parcial da veia porta.

**Métodos:**

Foram utilizados 24 ratos, distribuídos em dois grupos: Sham Operated e Hipertensão Portal. A ligadura parcial da veia porta foi realizada apenas no grupo Hipertensão Portal e, após 1/6/24 h e 3/5/14 dias, os animais foram eutanasiados. Foi feita a análise imunoistoquímica para quantificar a expressão da enzima corada, utilizando o programa ImageJ.

**Resultados:**

No grupo Hipertensão Portal, o miocárdio expressou percentuais entre 4,6-6% de área marcada, enquanto que no grupo Sham Operated variou entre 4-5%, sem significância estatística. Apenas no grupo Hipertensão Portal, a porcentagem corada pela enzima seguiu padrão crescente nas primeiras 6 h e decrescente após 24 h.

**Conclusão:**

A expressão da noradrenalina no miocárdio de ratos durante as primeiras duas semanas após a ligadura parcial da veia porta, tendo como marcador a enzima tirosina hidroxilase, não apresentou diferenças entre grupos ao longo do tempo.

## INTRODUCTION

The portal vein is formed by the union of superior mesenteric and splenic veins, and its tributaries include gastric and pancreatoduodenal veins. It extends to the hepatic hilum, and it is divided into right and left hepatic veins. It has a segmental intrahepatic distribution, accompanying the hepatic artery^3^ ^,^ ^12^. The liver receives a total blood flow about 1,200 ml/min, which represents approximately 25% of the cardiac output. It *receives*a dual*blood supply:* 25% of the volume comes from the hepatic artery, and 75% from the portal vein^3^.

The portal venous system has two important hemodynamic characteristics, which are the high blood flow with low resistance and low pressure. In adults, the portal pressure is approximately 7 mmHg^12^. It is directly related to resistance and blood flow, according to Ohm’s law (ΔP=Q x R)^4^ ^,^ ^12^. The ΔP corresponds to the portal vein pressure gradient (difference between portal pressure and inferior cava vein pressure), Q to portal blood flow and R to flow resistance^1^. Portal hypertension (PH) is the clinical syndrome usually secondary to intrahepatic or extrahepatic obstruction of the portal flow, in which the increased portal blood flow resistance *is the primary factor*in the pathophysiology of portal hypertension^12^.

PH is classified as pre-hepatic (eg. portal vein/splenic thrombosis), intra-hepatic (eg. cirrhosis) and post-hepatic (eg. hepatic vein/inferior cava vein thrombosis or congestive heart failure). The most common cause of PH is cirrhosis, whose increased resistance is primarily caused by distortion of the liver architecture (fibrosis and regenerative nodules). It is worth remembering that about one-third of the increase in resistance is due to intra-hepatic vasoconstriction, which can be vasodilated^2^. Due to the fact that the portal system is a set of veins that anastomose in the same place, whenever there is an obstruction, there will be increased pressure and development of collateral circulation, such as esophagogastric varices, perpetuating the development of splanchnic hyperdynamic circulation^3^ ^,^ ^4^.

This vasodilation and reduction of systemic vascular resistance leads to reduction of the effective arterial blood volume and activates the sympathetic nervous system, renin-angiotensin-aldosterone system and increases the release of vasopressin and endothelin-1, causing sodium retention and water. The consequences are increased plasma volume, cardiac output and heart rate, and decreased renal blood flow, hypotension, and more retention of fluid and water. Once hyperdynamic circulation is established, it can increase portal flow and cause further damage to the portal pressure. The portal hypertension and portosystemic shunt become an enclosed vicious cycle^18^.

Vasodilators - particularly nitric oxide and endothelins - appear to play a central role in circulatory derangement and contribute to the mechanism of splanchnic vasodilation^18^. In addition, endotoxins probably induce the production of prostacyclins, which also contribute to this process^6^. Sympathetic atrophy also occurs in the splanchnic area, due to the high levels of vasodilators, such as nitric oxide, and the reduction of vascular reactivity to vasoconstrictors^17^. In cirrhotic patients, in contrast, there is less production of nitric oxide in hepatic microvasculature, which also contributes to the hyperdynamic circulation^16^. Thus, splanchnic vasodilation appears to be the initial hemodynamic event following the increase in portal pressure, and also the trigger of subsequent hemodynamic changes, in which nitric oxide seems to be the main vasodilator involved^14^.

The portal vein partial ligation model (PVPL)^11^ has been widely used in the study of the pathophysiology of PH, because it reproduces all stages and hemodynamic changes in a well established sequence of events, making it possible to predict chronobiologically the changes that lead to hyperdynamic circulation^9^. Studies suggest that the excessive endothelial production of nitric oxide is directly related to the mesenteric vasoconstriction that occurs early after PVPL, and this vasoconstriction is a myogenic reflex to the acute increase of portal pressure and probably to vasoconstrictors. Right after the PVPL, there is an up-regulation of the genes related to adrenergic neurotransmission, and lately there is a down-regulation of the expression of these adrenergic genes and increased nitric oxide synthesis by the activation of NO synthetase, with characteristic mesenteric vasodilatation of the rats submitted to PVPL; this contributes to the state of hyperdynamic circulation and leads to the other complications of PH^5^.

The activation of such neurohumoral axis and the consequent hyperdynamic circulation of PH may lead to cardiac morphological and functional modifications, as the increase of the left and right atria and the increase of the diastolic diameter of the right ventricle. This can be interpreted as a cardiac hemodynamic adaptation to peripheral circulation changes, such as preload increase. Changes in diastolic function are frequently reported in these patients, and their presence is considered an early marker of cardiac injury^15^.

To*date*,*no experimental studies* have quantified adrenergic expression in the myocardium in the early stages of PH development.

The aim of this study was to analyze the expression of noradrenaline in the myocardium of rats during the first two weeks after PVPL, using the enzyme tyrosine hydroxylase (TH) as the marker^10^.

## METHODS

The experiment was conducted at Faculdade Evangélica do Paraná (FEPAR) and the Institute of Medical Research (IPEM), Curitiba, PR, Brazil. The research project was approved by the Research Ethics Committee of the Evangelical Beneficent Society of Curitiba (1408/2016). The procedures were in agreement with the ones recommended by the Ethical Committee on the Use of Animals of FEPAR.

Twenty-four male Wistar rats weighing between 200-300 g, from the Animal Hospital of the Paraná Institute of Technology, were maintained in the IPEM biotery in plastic boxes of 47x34x18 cm lined with a 12-h light/dark cycle and 22 ± 2° C temperature. The animals were treated daily with filtered water and appropriate ration administered freely.

**FIGURE 1:**
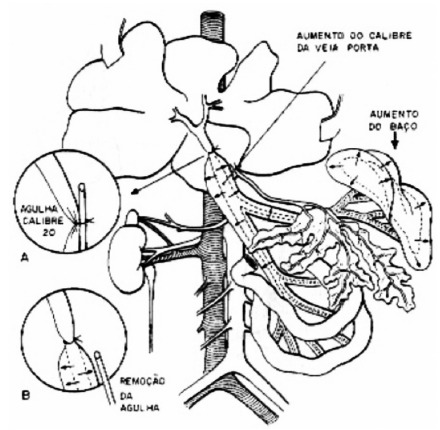
Portal vein partial ligation model (PVPL)

They were divided into two groups: Sham Operated, group submitted to the simulation of the operation, without the PVPL; and PVPL. A mixture of xylazine hydrochloride 10 mg/kg and ketamine hydrochloride 90 mg/kg intraperitoneally was used to anesthetize the animals. After anesthesia, the surgical intervention began with tricotomy and disinfection of the abdominal region, followed by a medium ventral laparotomy. The gut was gently exposed over a gauze humidified with saline and the portal vein was isolated. A 20 G needle was placed over the portal vein and both joined by a 3.0 silk thread. After the portal vein stenosis, the needle was gently removed. It was certified that there was no portal vein thrombosis during this manipulation^13^. The abdominal’s *skin*closure was done by simple *interrupted*suture technique, while peritoneum and abdominal muscle layer *were closed with continuous*suture. The Sham Operated underwent the same procedure, but they had their portal vein only manipulated. Figure 1 represents the schematic model of PVPL, established by Sikuler et al^13^.

After 1, 6 and 24 h, and 3, 5 and 14 days after the procedure, the rats were euthanized with an *overdose*of*anesthetic*agents (the same substances used in the pre-procedure anesthesia) by*intraperitoneal*injection. After euthanasia, the heart was removed, fixed in 10% formaldehyde dissolved in 0.1M PBS and pH 7.4 and the tissue was processed by conventional histological techniques. The fragments were embedded in paraffin and were cut in transverse sections. They were submitted to immunohistochemical analysis for TH, and myocardium (left ventricle) microscopic images were captured at 40x magnification. The captures were recorded in 24-bit and, with ImageJ software, which transformed them into 8-bit blue color. After the “threshold” command, followed by the “measure” command, the images were processed and the percentages of areas stained by the TH was quantified for later statistical analysis.

### Statistical analysis

The data collected were submitted to the software GraphpadInstat, version 3.0 for Windows XP2000, using the Wilcoxon test, adopting a significance level of 5% (p<0.05).

## RESULTS

**FIGURE 2:**
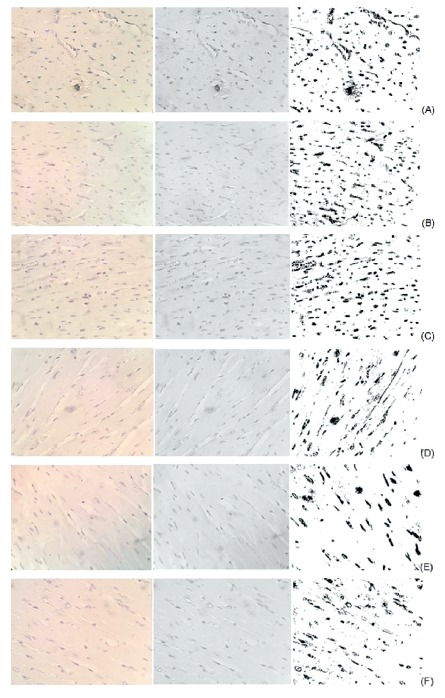
Myocardium in 1 h (A), 6 h (B) e 24 h (C) (40x)

**FIGURE 3:**
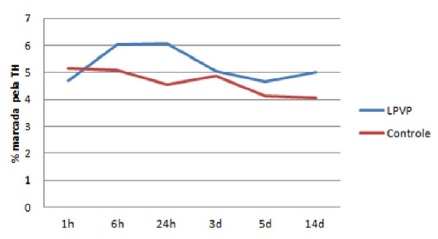
Myocardium in 3 (D), 5 (E) and 14 (F) days (40x)

Twenty-four rats, 12 PVPL and 12 Sham Operated participated in the experiment, and the samples were obtained at 1, 6 and 24 h and 3, 5 and 14 days. In Figure 2, the myocardial photomicrographs obtained after 1 h (A), 6 h (B) and 24 h (C) can be observed and in Figure 3 the 3 (D), 5 (E) and 14 (F) days of PVPL, at 24-bit, 8-bit resolutions and after the threshold command, respectively. The darker areas represent TH staining.

The percentage of area marked by TH was calculated by applying the “threshold” command followed by “measure” and the percentages of areas stained ​​are expressed in Table 1. The myocardium expressed percentages ranging from 4-5.1% in the Sham Operated group, and 4.6-6.8% in the PVPL group. There was no significant difference between groups (p <0.05). However, it is interesting to observe the different patterns of curves of the percentage stained by TH over time between groups, best seen in Figure 4.

**TABLE 1:** Area marked by TH in each group (%)

	Sham Operated	PVPL
1h	5,143	4,685
6h	5,065	6,020
24h	4,544	6,823
3d	4,886	5,045
5d	4,129	4,655
14d	4,042	5,020

**FIGURE 4:** Area marked by TH over timer (%)

## DISCUSSION

Many studies seek to elucidate the role of the autonomic nervous system in PH. Despite the increase systemic levels of catecholamines, it is known that this overactivity of the sympathetic nervous system is not homogeneous, since there are organs and tissues in which this overactivity has not been verified. The mesenteric vessels, for example, there is an important down-regulation of the genes related to adrenergic neurotransmission in the superior mesenteric artery after PVPL, accompanied also by regression/atrophy of sympathetic innervation throughout the mesenteric vascular territory. However, this nervous atrophy is not present in other vascular beds, such as the renal arteries. Mesenteric adrenergic down-regulation can be interpreted as a local consequence of PH, that may contribute to aggravate splanchnic vasodilation, which is responsible for generalized sympathetic hyperactivity, especially in muscles and kidneys^9^.

The available experimental evidences do not allow for a definitive conclusion about the importance of the sympathetic nervous system in the development of cardiac hypertrophy^7^. Studies suggest that increased markers of sympathetic innervation may be a common feature of early stages of compensated cardiac hypertrophy, regardless of the time. Sympathetic neural mechanisms do not seem to play a stimulating or trophic role in the hypertrophic process. On the other hand, they appear to be a secondary event, suggesting a possible stimulatory influence of sympathetic cardiac nerves over hypertrophied myocardium^8^. However, it is also known that norepinephrine and acetylcholine are depleted with the progression of manifest heart failure. This depletion causes less support for cardiac output in response to sympathetic nerve activation^19^. Based on these principles, the PVPL would present both inherent sympathetic hyperactivity - consequent to PH - and the stimulation of the sympathetic nervous system by induced cardiac hypertrophy. 

In this experiment, the TH immunohistochemistry in the myocardium after 1, 6, 24 h and 3, 5 and 14 days of PVPL was evaluated in order to identify the behavior of the sympathetic nervous system at the cardiac level in the different stages of PH. The PVPL model reproduces all systemic and hemodynamic changes detected in PH and the state of hyperdynamic circulation: increased pressure and portal flow, appearance of port-systemic shunts, splanchnic vasodilation with reduction of arterial and splanchnic flow resistance, systemic vasodilation with hypotension, reduction of peripheral resistance and increase in cardiac output. This model is very homogeneous, reproducible and with excellent chronobiological prediction, which elucidates the sequence of events involved in hyperdynamic circulation. The port-systemic shunt is detected after two days of PVPL and the percentage of portal blood inflow diverted to collaterals approaches 100% after one week. The circulation becomes hyperdynamic 4-5 days after PVPL and, one week after the procedure, the rats present a full range of PH changes^9^.

In this study, the myocardium expressed percentages that varied from 4 to 5.1% in the Sham Operated group, and from 4.6 to 6.8% in the PVPL group. Although there was no significant difference between the groups, it is interesting to observe the different patterns of growth curves of the *percentage*area*stained* by TH over time. In the PVPL group, there was an elevation in the first 6 h, remained stable until the end of the first 24 h, and then presented a decreasing pattern until the 5^th^ day. After the 5^th^ and until the 14^th^ day, the percentage returned to levels similar to those at the beginning of the experiment. This pattern was not observed in the Sham Operated group, whose levels maintained stable during the 14 days of experiment. Analyzing the chronobiological prediction of PVPL, the return of the *percentage*area*stained* after the 5^th^ to the 14^th^ day may be related to the beginning of the hyperdynamic circulation, predicted in this model from the 4^th^ to the 5^th^ day. Similarly to the mesenteric arterial bed, in which there is an initial sympathetic up-regulation with subsequent down-regulation, there may be in the myocardium similar mechanisms associated. However, further studies are needed to understand why the curves were so discrepant between the groups over the 14 days experiment.

## CONCLUSION

The expression of noradrenaline in rat myocardium during the first two weeks after partial ligation of the portal vein, with tyrosine hydroxylase as marker, did not show differences between groups over time.
